# Comparative polygenic predispositions of treatment-resistant depression in East Asian and European populations

**DOI:** 10.1038/s41386-025-02242-9

**Published:** 2025-09-19

**Authors:** Chi-Fung Cheng, Wei-Yi Kao, Mei-Chen Lin, Mei-Hsin Su, Chi-Shin Wu, Chun-Chieh Fan, Shi-Heng Wang

**Affiliations:** 1 National Center for Geriatrics and Welfare Research, National Health Research Institutes, Zhunan, Taiwan; 2 Department of Psychiatry, Virginia Institute for Psychiatric Behavioral Genetics, Virginia Commonwealth University, Richmond, VA USA; 3 Department of Psychiatry, National Taiwan University Hospital, Yunlin Branch, Yunlin, Taiwan; 4 Center for Population Neuroscience and Genetics, Laureate Institute for Brain Research, Tulsa, OK USA; 5 Department of Radiology, School of Medicine, University of California San Diego, La Jolla, CA USA; 6 Department of Medical Research, China Medical University Hospital, China Medical University, Taichung, Taiwan

**Keywords:** Genetic markers, Depression, Genetics research

## Abstract

Large-scale genetic studies of treatment-resistant depression (TRD) have been performed majority on European ancestry cohorts, potentially missing important population-specific biological insights. Understanding the genetic predisposition for TRD across populations could provide insights for etiologic heterogeneity. Conducting a cohort study of 106,796 unrelated participants using Taiwan Biobank (TWBB), we investigated the association of polygenic score (PGS) with the development of TRD among patients with depression and explore the concordance of the PGS association between East Asian and European populations. Three binary outcomes were defined, including TRD vs. non-major depressive disorder (MDD), treatment responsive MDD (trMDD) vs. non-MDD, and TRD vs. trMDD. Six PGSs belong to personality domains (nervous, worry, guilty feelings, neuroticism, tense, and worry embarrassment) and compulsive PGS were associated with TRD vs. trMDD in TWBB. The pattern of association was consistent across TRD definitions with different dose, duration, and interrupted window for antidepressant treatment. The estimated strength of PGS association in TWBB is consistent with that in All of US (AoU) (meta-analytic R^2^ was 68% for TRD vs. non-MDD, 81% for trMDD vs. non-MDD, and 77% for TRD vs. trMDD). PGSs for temperament and education were associated with TRD vs. trMDD in AoU but not in TWBB. There was a moderate to high trans-ancestry concordance for the genetic estimates with TRD, while PGS association for specific traits were not transferable between East Asian and European populations. Genetic research across ancestries and geographic regions is crucial to learn population-specific etiology.

## Introduction

Major depressive disorder (MDD) is believed to have a genetic etiology. A large-scale genome-wide association study (GWAS) of European populations [1] and a modest-scale GWAS of East Asian populations [2] have been conducted for MDD, and MDD loci were not transferable across ancestries and geographic regions while there was a moderate trans-ancestry genetic correlation [2].

Treatment-resistant depression (TRD), a trait of occurrence of poor antidepressant responses, is considered to have a genetic component [3]. However, most previous molecular genetic studies for TRD have been conducted in European ancestry cohorts [4–7]. Conducting large-scale genetic research in individuals of diverse ancestries is crucial to learn novel biological insights pertinent to specific populations and needed to explore potential population difference in genetic architecture. Polygenic scores (PGS) have been used to explore etiology of TRD in European ancestry cohorts [4–7], however, large-scale PGS research for TRD still lacks in East Asian ancestry cohorts. Understanding the PGS association of TRD across populations could provide insights for etiologic heterogeneity.

Using a large collection of community samples from the Taiwan Biobank (TWBB) of East Asian populations with linkage to insurance databases, this study aimed to investigate the association of PGS for five domains, including personality, temperament, education and cognition, sleep patterns, and psychiatry, with TRD. A series of sensitivity analyses for the TRD definitions was performed to evaluate the robustness. In addition, we explored the concordance of the PGS association with TRD between this TWBB study and a recent study using All of Us (AoU) [8] of European populations.

## Methods and materials

### Taiwan Biobank

The TWBB [9–12], the largest government-supported biobank in Taiwan since 2012, recruits community-based participants aged 30–70 years. This study conducted a cohort study using TWBB with linkage to National Health Insurance Research Database (NHIRD) to retrieve ICD diagnoses and history of medication treatment. The study described in this paper was approved by the Institutional Review Board (IRB) of the Central Regional Research Ethics Committee of the China Medical University, Taichung, Taiwan (CRREC-108-30). The TWBB obtained informed consent from all participants for research use of the collected data and samples and the linkage with the NHIRD.

### Genotyping and quality control

This study comprised 131,048 individuals with genome-wide genotyping using the custom Taiwan Biobank chips ran on the Axiom Genome-Wide Array Plate System (Affymetrix, SantaClara,CA, USA); detailed quality control process is detailed in our previous study [13, 14]. Before imputation, variants with a call rate < 5%, minor allele frequency < 0.001, and deviation from Hardy-Weinberg equilibrium with *p* < 1E−06 were excluded. We used the 504 EAS panel from 1000 Genomes Project [15] and the 973 TWBB panel from whole-genome sequencing in TWBB participants as the reference panel to impute the genotypes with IMPUTE2. After imputation, 12,601,684 variants with imputation info > 0.7 were selected for subsequent PGS calculation.

After excluding duplicated samples, non-East Asian samples, samples with a missing rate of more than 2%, or heterozygosity outliers (exceeding 5 standard deviation), 128,775 samples remained. Principal components analysis was performed to account for population stratification. To remove cryptic relatedness, one of the study participants was removed if pair-wise participants with PI-HAT > 0.1875. Totally, 106,796 unrelated participants were included for subsequent PGS calculation and linked to NHIRD.

### PGS

We calculated 41 PGSs for five domains: personality (19 PGS), temperament (6 PGS), education and cognition (2 PGS), sleep patterns (3 PGS), and psychiatric disorders (11 PGS). Detailed information for the PGS was showed in Supplementary Table 1. We derived the PGS using PRS-CS [16], a polygenic prediction method inferring posterior effect sizes of susceptibility variants by utilizing a high-dimensional Bayesian regression framework and continuous shrinkage priors on susceptibility variant effect sizes, which have been shown robust to diverse underlying genetic architectures. We used the 1000 Genomes Project phase 3 EUR samples as LD reference panel (–ref_dir= /ldblk_1kg_eur; matching the ancestry of majority of discovery samples) and fixed global shrinkage parameter 0.01 (–phi=1e-2). The PGS was calculated using PLINK (https://www.cog-genomics.org/plink/) and normalized for subsequent analyses.

### ICD diagnoses in National Health Insurance Research Database

After excluding patients with schizophrenia (ICD9 code: 295; ICD10 code: F20, F25) (*n* = 668) or bipolar disorder (ICD9 code: 296.0–296.1, 296.4–296.8; ICD10 code: F30, F31, F34.0) (*n* = 1476), 13,654 individuals have MDD (ICD9 code: 296.2, 296.3, 300.4, 311; ICD10 code: F32, F33, F34.1), and the remaining 90,998 individuals have no MDD (non-MDD). Among 11,563 newly onset MDD since 2000, 9971 patients ever received antidepressant treatment.

### TRD

There is no standard for estimating treatment outcomes in real-world data, some recommendations for defining TRD has been summarized in a recent review [17]. We defined TRD as at least two changes in adequate antidepressant treatment trial within a period of 2 years after antidepressant treatment. Different scenarios for the minimum duration and the interrupted window were considered for an adequate antidepressant treatment trial. A meta-analysis of clinical trials suggested that nonresponse can be reliably predicted within 2 weeks after initiating an antidepressant treatment [18]. A STAR*D trial suggested that the average time to response is above 4 weeks [19]. Two GWASs applied a minimum antidepressant trial duration of 6 weeks [4, 20]. Side effects might lead to earlier switching medications [17]. In this study, we defined the minimum duration as ≥ 2, 4, or 6 weeks; defining the minimum antidepressant trial duration as 2 weeks identified largest TRD cases. A previous TRD study in Taiwan considered that an interrupted window of <14 days was allowed, in which antidepressant treatment was stopped and restarted within a 14-day period; all discontinuation intervals lasting <14 days were considered part of the same treatment trial [21]. We defined the interrupted window of < 14, 21, 28, or infinite days. These different scenarios identified TRD cases ranged 440–1646, and the remaining 8325-9531 were defined treatment responsive MDD (trMDD).

After applying additional criterion of minimal dose for antidepressant, among 7526 patients ever received antidepressant treatment ≥ minimal dose (minimal dose for each antidepressant was detailed in Supplementary Table 2) [22], the number of TRD cases ranged 91–548. Defining TRD by antipsychotics add on, 165 patients were defined TRD.

Samples sizes for different TRD definitions are detailed in Supplementary Table 3. We used the TRD criterion, identifying most TRD cases (*n* = 1646), of more than two distinct trial ≥ 2 weeks of any-dose antidepressant medication and interrupted window of < 14 days as the main analyses, and other criterion as sensitivity analyses.

### Statistical analyses

We tested the association of PGS with three binary outcomes: TRD vs. non-MDD, trMDD vs. non-MDD, and TRD vs. trMDD. The significance of each PGS was evaluated using logistic regression models adjusted for sex, age at last follow-up in the NHIRD, and 20 principal components. We set the significance level at *p* = 0.05 and evaluate the robustness of the PGS association by performing sensitivity analyses for different TRD definitions. For each binary outcome, 41 PGSs were tested, hence the Bonferroni-corrected significance level was set at 0.05/41 = 0.0012. Nominal significance was defined if 0.0012 < *p* < 0.05 for an association test. We examined the within-TWBB consistency of the estimates for PGS across different TRD definitions by calculating R^2^ using multilevel meta-regression with R package *metaphor* and the overlap of significant PGS associations. The concordance of the PGS association with TRD between this TWBB study and a recent AoU study (using the version 7 data release of the AoU) was evaluated using random-effect meta-regression.

## Results

### PGS associations in TWBB

**Fig. 1:**
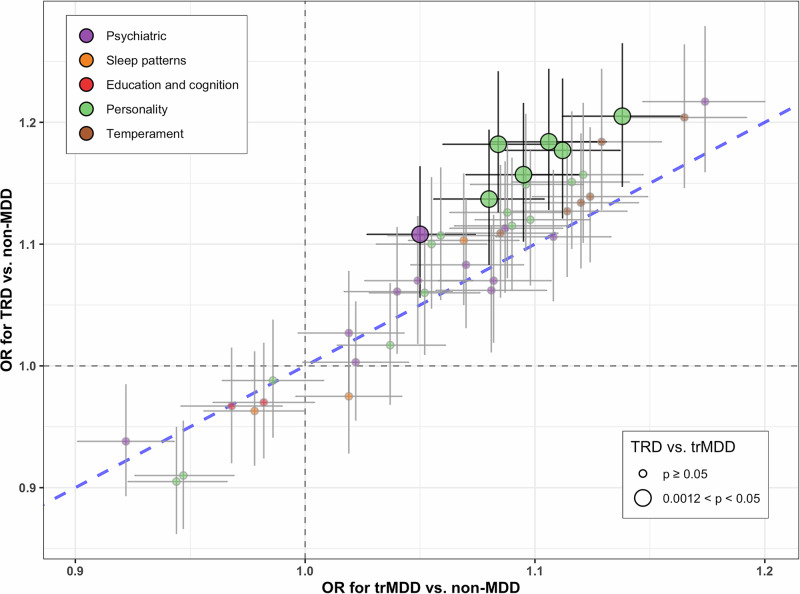
Association of PGS with treatment-resistant MDD (TRD) and treatment-responsive MDD (trMDD). The scatter plot of the odds ratio (OR), with the corresponding 95% confidence interval, between PGS and TRD vs. non-MDD against the OR between PGS and trMDD vs. non-MDD. PGSs associated with TRD vs. trMDD with nominal significance are showed in solid colors. TRD definition: within an observation period of 2 years after initiating antidepressant treatment, at least two changes in treatment regimen, with an adequate trial of antidepressant prescription duration ≥ 2 weeks and interrupted window < 14 days. Sample size: 1646 TRD, 8325 trMDD, and 90,998 non-MDD.

The distributions of demographic characteristics in 1646 TRD, 8325 trMDD, and 90,998 non-MDD individuals are presented in Supplementary Table 4. The associations of 41 PGSs with TRD and trMDD are summarized in Fig. 1; 27 PGSs are associated with the risk of being TRD vs. non-MDD, and 33 PGSs are associated with the risk of being trMDD vs. non-MDD. Generally, the estimated strength of association is larger in TRD vs. non-MDD than in trMDD vs. non-MDD.

**Fig. 2:**
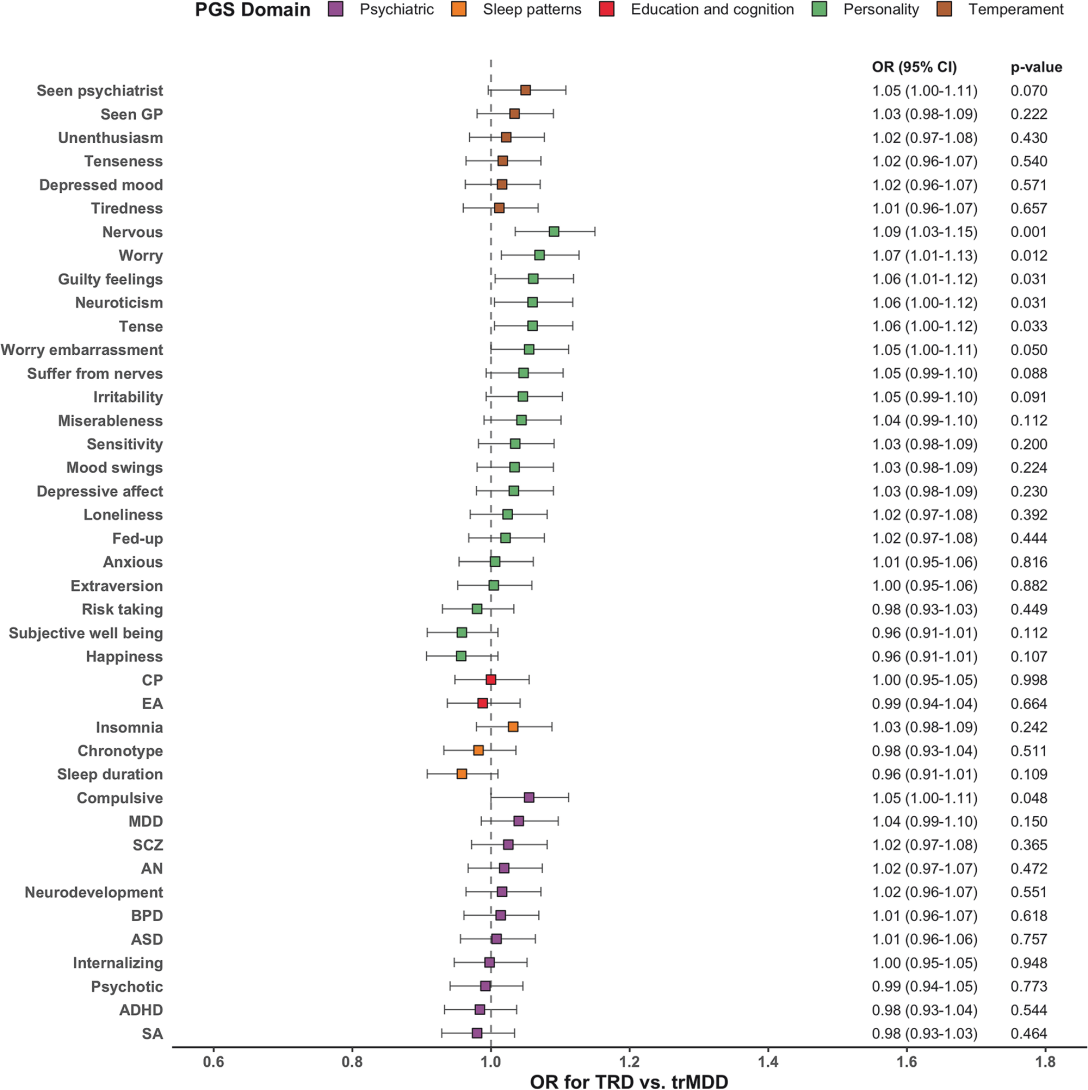
Forest plot of the odds ratio (OR) with the corresponding 95% confidence interval (CI) between PGS and treatment-resistant MDD (TRD) vs. treatment-responsive MDD (trMDD). TRD definition: within an observation period of 2 years after initiating antidepressant treatment, at least two changes in treatment regimen, with an adequate trial of antidepressant prescription duration ≥ 2 weeks and interrupted window < 14 days. Sample size: 1646 TRD, 8325 trMDD, and 90,998 non-MDD. CP cognitive performance, EA educational attainment, MDD major depressive disorder, SCZ schizophrenia, AN anorexia nervosa, BPD bipolar disorder, ASD autism spectrum disorder, ADHD attention deficit hyperactivity disorder, SA suicide attempt.

The forest plot of the odds ratio (OR) with 95% confidence intervals (CI) between PGSs and being TRD vs trMDD is demonstrated in Fig. 2. The PGSs belong to personality and psychiatric domains have stronger associations with TRD than with trMDD. Regarding the personality domain, PGSs for nervous (OR = 1.09 per one standard deviation increase in PGS, 95% CI: 1.03–1.15, *p*-value = 0.0013), worry (OR = 1.07, 95% CI: 1.01–1.13, *p*-value = 0.0122), guilty feelings (OR = 1.06, 95% CI: 1.01–1.12, *p*-value = 0.0306), neuroticism (OR = 1.06, 95% CI: 1.00–1.12, *p*-value = 0.0315), tense (OR = 1.06, 95% CI: 1.00–1.12, *p*-value = 0.0332), and worry embarrassment (OR = 1.05, 95% CI: 1.00–1.11, *p*-value = 0.0496) were associated with increased likelihood of being TRD with nominal significance. PGS for compulsive of psychiatric domain was associated with 5% (95% CI: 1.00–1.11, *p*-value = 0.0479) increased likelihood of being TRD with nominal significance.

### Sensitivity analyses

Across 24 combinations of TRD definitions with different dose, duration, and interrupted window for antidepressant treatment, the estimated strength of association is consistent (forest plots in Supplementary Fig. 1; meta-regression R^2^ and overlap of significant PGS associations in Supplementary Table 5). Defining TRD as more than two distinct trial ≥ 2/4 weeks of any-dose and ≥ 2 weeks of minimal-dose antidepressant medication, regardless of interrupted window, have meta-analytic R^2^ above 90% for TRD vs. non-MDD, approximately 100% for trMDD vs. non-MDD, and above 55% for TRD vs. trMDD compared to the primary results. All combinations of TRD definitions have the same set of 33 PGSs shown to be significantly associated with trMDD vs. non-MDD in the primary analyses. Scenarios with TRD cases < 500 have limited statistical power for identifying significant PGS associations with TRD vs. non-MDD and TRD vs. trMDD and limited overlapping significant PGS association with the primary results.

Regarding the seven PGSs associated with being TRD vs trMDD with nominal significance, sensitivity analyses showed consistent point estimates across different TRD definitions (see forest plots in Supplementary Fig. 1).

Defining TRD by antipsychotics add on produced distinctive results, and meta-analytic R^2^ was 57% for TRD vs. non-MDD and 5% for TRD vs. trMDD compared to the primary results (Supplementary Table 5). PGS for subjective well being belong to personality domain and PGSs for ADHD, AN, and BPD belong to psychiatric domain have stronger associations with TRD defined by antipsychotics than with trMDD (Supplementary Fig. 2). PGS for subjective well being (OR = 0.76, 95%CI: 0.65–0.89, *p*-value = 0.0006) was significantly associated with decreased likelihood of receiving antipsychotics add on, and PGSs for ADHD (OR = 1.25, 95%CI: 1.07–1.46, *p*-value = 0.0043), AN (OR = 1.20, 95%CI: 1.03–1.40, *p*-value = 0.0217), and BPD (OR = 1.17, 95%CI: 1.00–1.37, *p*-value = 0.0451) were associated with increased likelihood of receiving antipsychotics with nominal significance (Supplementary Fig. 3).

### Compared with AoU

**Fig. 3:**
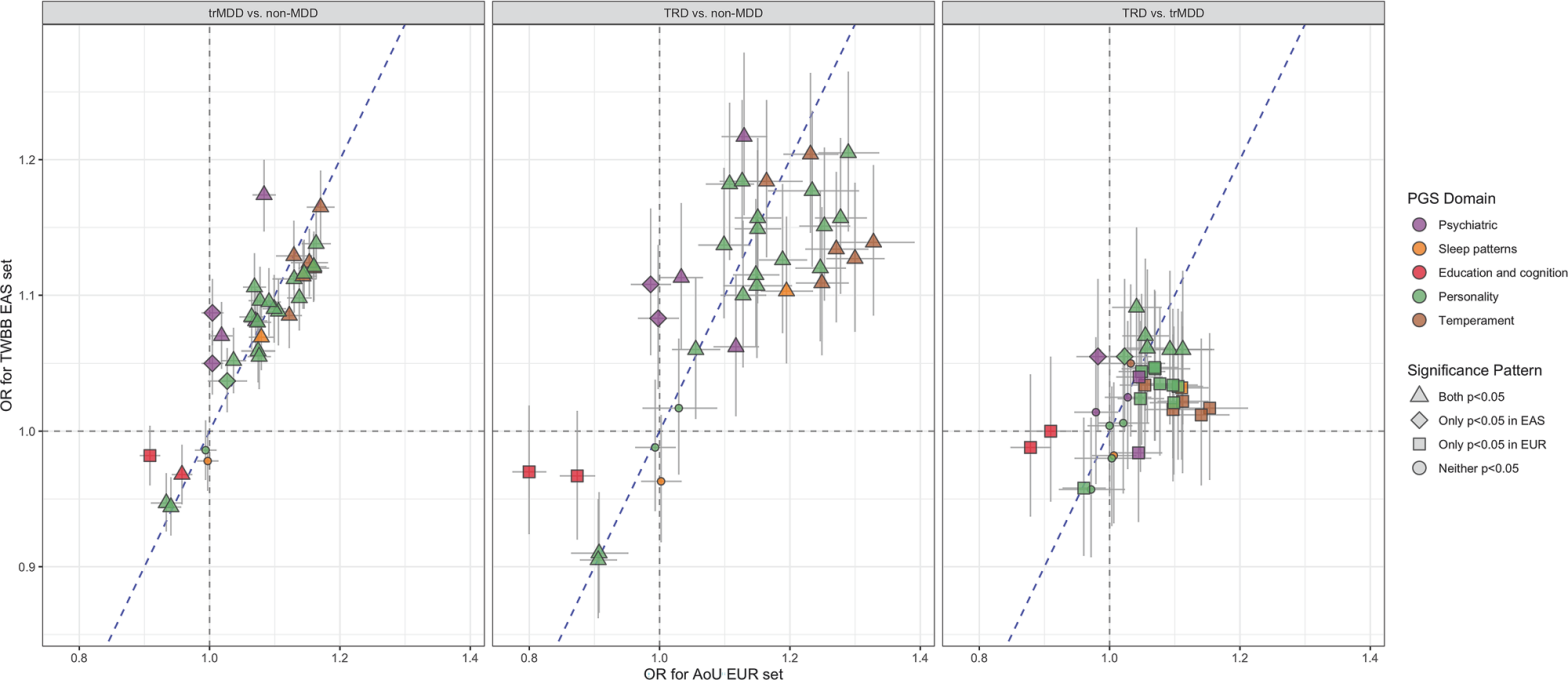
The scatter plot of the odds ratio (OR), with the corresponding 95% confidence interval, for PGS in Taiwan Biobank of East Asian populations against the OR for PGS in All of US of European populations.

Generally, the OR in TWBB is consistent with that in AoU (see forest plots in Supplementary Fig. 4), and meta-analytic R^2^ was 68% for TRD vs. non-MDD, 81% for trMDD vs. non-MDD, and 77% for TRD vs. trMDD. The scatter plots of the OR in TWBB against the OR in AoU are shown in Fig. 3. PGSs of personality and temperament domains and PGS for insomnia were associated with trMDD vs. non-MDD and TRD vs. non-MDD in both TWBB and AoU. PGSs belong to personality domains were associated with TRD vs. trMDD in both TWBB and AoU. However, PGSs for temperament and education and cognition domains and PGS for insomnia, MDD, and ADHD were associated with TRD vs. trMDD in AoU but not in TWBB. PGSs for worry embarrassment and compulsive were associated with TRD vs. trMDD in TWBB only.

## Discussion

We performed the largest molecular genetic study (to our knowledge) in patients with MDD of East Asian populations, used polygenetic score to assess an individual’s genetic predisposition to various traits and diseases, and observed that personality was genetically associated with increased risk of developing TRD. These findings were highly consistent across different TRD definitions. A moderate to high concordance of the genetic estimates with TRD existed between TWBB and AoU, while some PGS associations were not transferable between East Asian and European populations, e.g., the effect of PGSs for temperament and education and cognition domains on TRD demonstrated in AoU was not evidenced in TWBB.

Our results, genetic liability for psychiatric disorders was associated with incidence of MDD but not associated with being TRD vs trMDD, echoed findings in the AoU [8] and UK cohorts [4, 5**]**. Psychosocial factors, e.g., personality and education, might contribute to the treatment and progression of MDD. The role of nervous-related personality in the incidence and treatment response of MDD was evidenced in both TWBB and AoU, and the trans-ancestry finding suggested the shared genetic etiology between the personality trait and severity and progression of MDD [23**–**25]. In TWBB, although the associations of PGSs belong to personality domain with increased likelihood of being TRD did not reach Bonferroni-corrected significance in the main analyses, the point estimates should be unbiased. Furthermore, the point estimates were consistent across different TRD definitions in the sensitivity analyses and comparable with AoU. Meanwhile, the inconsistent results for the association of PGSs for education and cognition with TRD in AoU and TWBB suggested that nongenetic factors, such as cultural difference across ancestries and geographic regions, may play a role on the treatment for MDD and moderate genetic associations through gene-environment interactions.

The proportion of TRD with different scenarios was 4–16% in the patients with MDD recruited from Taiwan Biobank with linkage to insurance databases, which is comparable to the 11–15% in studies in Denmark and Sweden [26**–**28] and lower than the 20% reported in AoU [8] and 30% in the STAR*D trial [19]. Variations in TRD definitions and study samples as well as cultural and regional differences in psychiatric care practice can lead to differential estimates of TRD proportion. In these electronic health record- and registry-based studies, TRD was determined by drug prescription patterns, lacking symptom measures, medication adherence information, and non-pharmacological treatment. Misclassifications of TRD status is possible. However, our sensitivity analyses showed that the associations for TRD were robust across different scenarios for the minimum duration, the interrupted window, and the minimum dose for antidepressant.

Our study has some limitations. First, the information of medication adherence is missing, and misclassification of TRD possibly limits the power for association test. Second, PGS only considers common variants and captures a limited heritability of a trait. Most large-scale GWAS have been performed in individuals of European populations, with only a few in individuals of East Asian populations. Using European ancestry as discovery samples, the PGS prediction performance in our target samples of East Asian ancestry would be reduced, compared to the PGS prediction performance in the AoU, as genetic architecture differs across populations [29, 30]. In addition, the sample size in the TWBB is smaller than in the AoU. Therefore, the power of the PGS analyses would be lower in the TWBB than in the AoU. Further large-scale genetic research in individuals of diverse ancestries is needed to mitigate the health disparities that exist across the populations [31].

## Conclusions

This study suggested that polygenic liability for nervous-related personality was associated with the development of TRD among patients with MDD of East Asian populations and indicated that the common emotional process might be potential target for treatment strategies. There was a moderate to high trans-ancestry concordance for the genetic estimates with TRD, while PGS association for specific traits were not transferable between East Asian and European populations. Generalizing findings about genetic predisposition for TRD across populations should be cautious. These results indicated complexity and potential population difference in genetic architecture for TRD as well as the importance of genetic research across diverse ancestries geographic regions.

## Supplementary information

Supplementary tables and figures

## Data Availability

The NHIRD used in this study is held by the Taiwan Ministry of Health and Welfare and under controlled access. Researchers interested in accessing the data set can submit an application form to the Ministry of Health and Welfare requesting access. Taiwan Biobank data used in this study is under controlled access. Application to access can be made to the Taiwan Biobank.
