# Supplementary material for: Comparative polygenic predispositions of treatment-resistant depression in East Asian and European populations
Source: Neuropsychopharmacology. 2025 Sep 19;51(4):753–8. doi: 10.1038/s41386-025-02242-9 (PMC12932758; doi:10.1038/s41386-025-02242-9)
Supplement: Supplementary file 1 — Supplementary tables and figures [file 41386_2025_2242_MOESM1_ESM.docx]

Supplementary Table 1. Detailed information for the PGS.

| Domain | PGS | PMID | Year | Sample size | Ancestry | No. SNP |
| --- | --- | --- | --- | --- | --- | --- |
| Personality | Anxious | 31427789 | 2019 | 376411 | EUR | 149,777 |
|  | Depressive affect | 29942085 | 2018 | 357957 | EUR | 882,416 |
|  | Extraversion | 26362575 | 2016 | 63661 | EUR | 898,054 |
|  | Fed-up | 29500382 | 2018 | 266208 | EUR | 882,411 |
|  | Guilty feelings | 29500382 | 2018 | 265139 | EUR | 882,411 |
|  | Happiness | 31427789 | 2019 | 128677 | EUR | 882,411 |
|  | Irritability | 31427789 | 2019 | 369232 | EUR | 492,621 |
|  | Loneliness | 29500382 | 2018 | 267190 | EUR | 882,411 |
|  | Miserableness | 29500382 | 2018 | 267050 | EUR | 329,767 |
|  | Mood swings | 29500382 | 2018 | 265382 | EUR | 882,411 |
|  | Nervous | 31427789 | 2019 | 376368 | EUR | 882,411 |
|  | Neuroticism | 31427789 | 2019 | 312740 | EUR | 882,411 |
|  | Risk taking | 31427789 | 2019 | 372651 | EUR | 882,411 |
|  | Sensitivity | 29500382 | 2018 | 264144 | EUR | 882,411 |
|  | Subjective well being | 27089181 | 2016 | 298420 | EUR | 812,714 |
|  | Suffer from nerves | 29500382 | 2018 | 262321 | EUR | 882,411 |
|  | Tense | 31427789 | 2019 | 374129 | EUR | 882,411 |
|  | Worry | 29942085 | 2018 | 348219 | EUR | 662,477 |
|  | Worry too long after embarrassment | 31427789 | 2019 | 370660 | EUR | 882,411 |
| Temperament | Frequency of depressed mood in last 2 weeks | 31427789 | 2019 | 370017 | EUR | 882,411 |
|  | Frequency of tenseness / restlessness in last 2 weeks | 31427789 | 2019 | 371869 | EUR | 882,411 |
|  | Frequency of tiredness / lethargy in last 2 weeks | 31427789 | 2019 | 375053 | EUR | 882,411 |
|  | Frequency of unenthusiasm / disinterest in last 2 weeks | 31427789 | 2019 | 373833 | EUR | 882,411 |
|  | Seen a psychiatrist for nerves, anxiety, tension or depression | 31427789 | 2019 | 384700 | EUR | 882,411 |
|  | Seen doctor (GP) for nerves, anxiety, tension or depression | 31427789 | 2019 | 383771 | EUR | 882,411 |
| Education and cognition | Education | 35361970 | 2022 | 3037499 | EUR | 870,072 |
|  | Cognitive performance | 30038396 | 2018 | 1070751 | EUR | 869,465 |
| Sleep | Insomnia | 30800456 | 2019 | 386078 | Multi | 877,268 |
|  | Chronotype | 30696823 | 2019 | 345148 | Multi | 875,168 |
|  | Sleep duration | - 30846698 | 2019 | 446118 | EUR | 877,268 |
| Psychiatric disorders | Schizophrenia | 35396580 | 2022 | 161405 | Multi | 909,529 |
|  | Bipolar disorder | - 34002096 | 2021 | 413466 | EUR | 871,110 |
|  | MDD | - 30718901 | 2019 | 807553 | EUR | 907,330 |
|  | ASD | - 30804558 | 2019 | 46350 | EUR | 865,345 |
|  | ADHD | 36702997 | 2022 | 225534 | EUR | 855,107 |
|  | Anorexia nervosa | 31308545 | 2019 | 72517 | EUR | 852,848 |
|  | Suicide attempt | 34861974 | 2022 | 549743 | Multi | 825,898 |
|  | Psychotic | - 35513722 | 2022 | 1559358 | EUR | 722,282 |
|  | Compulsive | 35513722 | 2022 | 1559358 | EUR | 722,282 |
|  | Neurodevelopmental | 35513722 | 2022 | 1559358 | EUR | 722,282 |
|  | Internalizing | 35513722 | 2022 | 1559358 | EUR | 722,282 |

Supplementary Table 2. Minimal dose for antidepressants.

| ATC code | Drug | Minimum dose (mg) |
| --- | --- | --- |
| N06AA02 | imipramine | 150 |
| N06AA04 | clomipramine | 150 |
| N06AA09 | amitriptyline | 150 |
| N06AA12 | doxepin | 150 |
| N06AA16 | dosulepin | 75 |
| N06AA21 | maprotiline | 150 |
| N06AB03 | fluoxetine | 20 |
| N06AB04 | citalopram | 20 |
| N06AB05 | paroxetine | 20 |
| N06AB06 | sertraline | 50 |
| N06AB08 | fluvoxamine | 50 |
| N06AB10 | escitalopram | 10 |
| N06AG02 | moclobemide | 300 |
| N06AX01 | oxitriptan | 100 |
| N06AX03 | mianserin | 30 |
| N06AX05 | trazodone | 300 |
| N06AX09 | viloxazine | 200 |
| N06AX11 | mirtazapine | 15 |
| N06AX12 | bupropion | 300 |
| N06AX16 | venlafaxine | 150 |
| N06AX17 | milnacipran | 100 |
| N06AX21 | duloxetine | 60 |
| N06AX22 | agomelatine | 25 |
| N06AX26 | vortioxetine | 5 |

Supplementary Table 3. Sample size for different TRD definitions

| Dose, Period, Windows | TRD | trMDD | non-MDD |
| --- | --- | --- | --- |
| any; 2 wk; 14d | 1646 | 8325 | 90998 |
| any; 2 wk; 21d | 1623 | 8348 | 90998 |
| any; 2 wk; 28d | 1584 | 8387 | 90998 |
| any; 2 wk; infinite | 1208 | 8763 | 90998 |
| any; 4 wk; 14d | 1059 | 8912 | 90998 |
| any; 4 wk; 21d | 1056 | 8915 | 90998 |
| any; 4 wk; 28d | 1025 | 8946 | 90998 |
| any; 4 wk; infinite | 764 | 9207 | 90998 |
| any; 6 wk; 14d | 496 | 9475 | 90998 |
| any; 6 wk; 21d | 506 | 9465 | 90998 |
| any; 6 wk; 28d | 507 | 9464 | 90998 |
| any; 6 wk; infinite | 440 | 9531 | 90998 |
| min; 2 wk; 14d | 552 | 6974 | 90998 |
| min; 2 wk; 21d | 548 | 6978 | 90998 |
| min; 2 wk; 28d | 540 | 6986 | 90998 |
| min; 2 wk; infinite | 375 | 7151 | 90998 |
| min; 4 wk; 14d | 310 | 7216 | 90998 |
| min; 4 wk; 21d | 313 | 7213 | 90998 |
| min; 4 wk; 28d | 311 | 7215 | 90998 |
| min; 4 wk; infinite | 215 | 7311 | 90998 |
| min; 6 wk; 14d | 107 | 7419 | 90998 |
| min; 6 wk; 21d | 113 | 7413 | 90998 |
| min; 6 wk; 28d | 119 | 7407 | 90998 |
| min; 6 wk; infinite | 91 | 7435 | 90998 |
| min; Anti-psychotics | 165 | 7361 | 90998 |

Supplementary Table 4. Demographic characteristics.

|  | TRD | trMDD | non-MDD |
| --- | --- | --- | --- |
|  | n=1,646 | n=8,325 | n=90,998 |
| Sex, n (%) |  |  |  |
| Female | 1,139 (69.20%) | 6,089 (73.14%) | 55,591 (61.09%) |
| Male | 507 (30.80%) | 2,236 (26.86%) | 35,407 (38.91%) |
| Birth year, mean (SD) | 1964 (11.14) | 1964 (11.21) | 1967 (11.69) |
| Recruited age in TWBB, mean (SD) | 52.29 (10.73) | 52.13 (10.76) | 49.62 (11.18) |
| Last follow-up age in NHIRD, mean (SD) | 58.29 (11.14) | 58.15 (11.18) | 55.34 (11.67) |

Supplementary Table 5. Meta-regression R^2^ and overlap of significant associations across sensitivity analyses with different treatment-resistant depression (TRD) definitions, compared to the primary result.

| Definitions  Dose, Period, Windows | TRD vs. non-MDD | | | trMDD vs. non-MDD | | | TRD vs. trMDD | | |
| --- | --- | --- | --- | --- | --- | --- | --- | --- | --- |
|  | No. of significant PGS  (p<0.0012) | Overlapping with the primary result | R^2^ | No. of significant PGS  (p<0.0012) | Overlapping with the primary result | R^2^ | No. of significant PGS  (p<0.05) | Overlapping with the primary result | R^2^ |
| Primary result  any; 2 wk; 14d | 27 | Ref. | Ref. | 33 | Ref. | Ref. | 7 | Ref. | Ref. |
| any; 2 wk; 21d | 28 | 100.00% | 99.86% | 33 | 100.00% | 99.99% | 5 | 71.40% | 98.92% |
| any; 2 wk; 28d | 28 | 100.00% | 99.71% | 33 | 100.00% | 99.98% | 6 | 71.40% | 97.10% |
| any; 2 wk; infinite | 25 | 92.60% | 97.47% | 34 | 100.00% | 99.84% | 9 | 71.40% | 78.56% |
| any; 4 wk; 14d | 24 | 88.90% | 95.88% | 34 | 100.00% | 99.82% | 3 | 28.57% | 73.73% |
| any; 4 wk; 21d | 23 | 85.20% | 96.30% | 34 | 100.00% | 99.83% | 3 | 14.29% | 75.80% |
| any; 4 wk; 28d | 22 | 81.50% | 96.24% | 33 | 100.00% | 99.82% | 3 | 14.29% | 75.39% |
| any; 4 wk; infinite | 21 | 77.80% | 92.05% | 33 | 100.00% | 99.66% | 5 | 0.00% | 55.24% |
| any; 6 wk; 14d | 11 | 40.70% | 80.81% | 34 | 100.00% | 99.66% | 4 | 28.57% | 35.71% |
| any; 6 wk; 21d | 9 | 33.30% | 81.38% | 34 | 100.00% | 99.68% | 4 | 28.57% | 37.17% |
| any; 6 wk; 28d | 9 | 33.30% | 83.27% | 34 | 100.00% | 99.66% | 3 | 28.57% | 35.00% |
| any; 6 wk; infinite | 10 | 37.00% | 82.54% | 34 | 100.00% | 99.60% | 2 | 14.29% | 32.53% |
| min; 2 wk; 14d | 20 | 74.10% | 91.43% | 33 | 100.00% | 99.68% | 5 | 14.29% | 54.90% |
| min; 2 wk; 21d | 21 | 77.80% | 91.86% | 33 | 100.00% | 99.65% | 7 | 42.86% | 55.90% |
| min; 2 wk; 28d | 20 | 74.10% | 92.48% | 33 | 100.00% | 99.66% | 7 | 42.86% | 58.82% |
| min; 2 wk; infinite | 11 | 40.70% | 90.90% | 33 | 100.00% | 99.67% | 4 | 14.29% | 58.73% |
| min; 4 wk; 14d | 7 | 25.90% | 83.57% | 33 | 100.00% | 99.65% | 3 | 14.29% | 44.16% |
| min; 4 wk; 21d | 7 | 25.90% | 85.29% | 33 | 100.00% | 99.66% | 4 | 14.29% | 47.46% |
| min; 4 wk; 28d | 8 | 29.60% | 86.26% | 33 | 100.00% | 99.68% | 4 | 14.29% | 51.12% |
| min; 4 wk; infinite | 4 | 14.80% | 74.90% | 34 | 100.00% | 99.63% | 4 | 0.00% | 29.39% |
| min; 6 wk; 14d | 0 | 0.00% | 67.31% | 34 | 100.00% | 99.62% | 0 | 0.00% | 26.75% |
| min; 6 wk; 21d | 0 | 0.00% | 64.52% | 34 | 100.00% | 99.61% | 0 | 0.00% | 17.68% |
| min; 6 wk; 28d | 0 | 0.00% | 55.97% | 34 | 100.00% | 99.60% | 0 | 0.00% | 11.60% |
| min; 6 wk; infinite | 0 | 0.00% | 50.58% | 35 | 100.00% | 99.64% | 2 | 0.00% | 25.16% |
| min; Anti-psychotics | 2 | 3.70% | 57.28% | 33 | 100.00% | 99.45% | 4 | 0.00% | 4.88% |


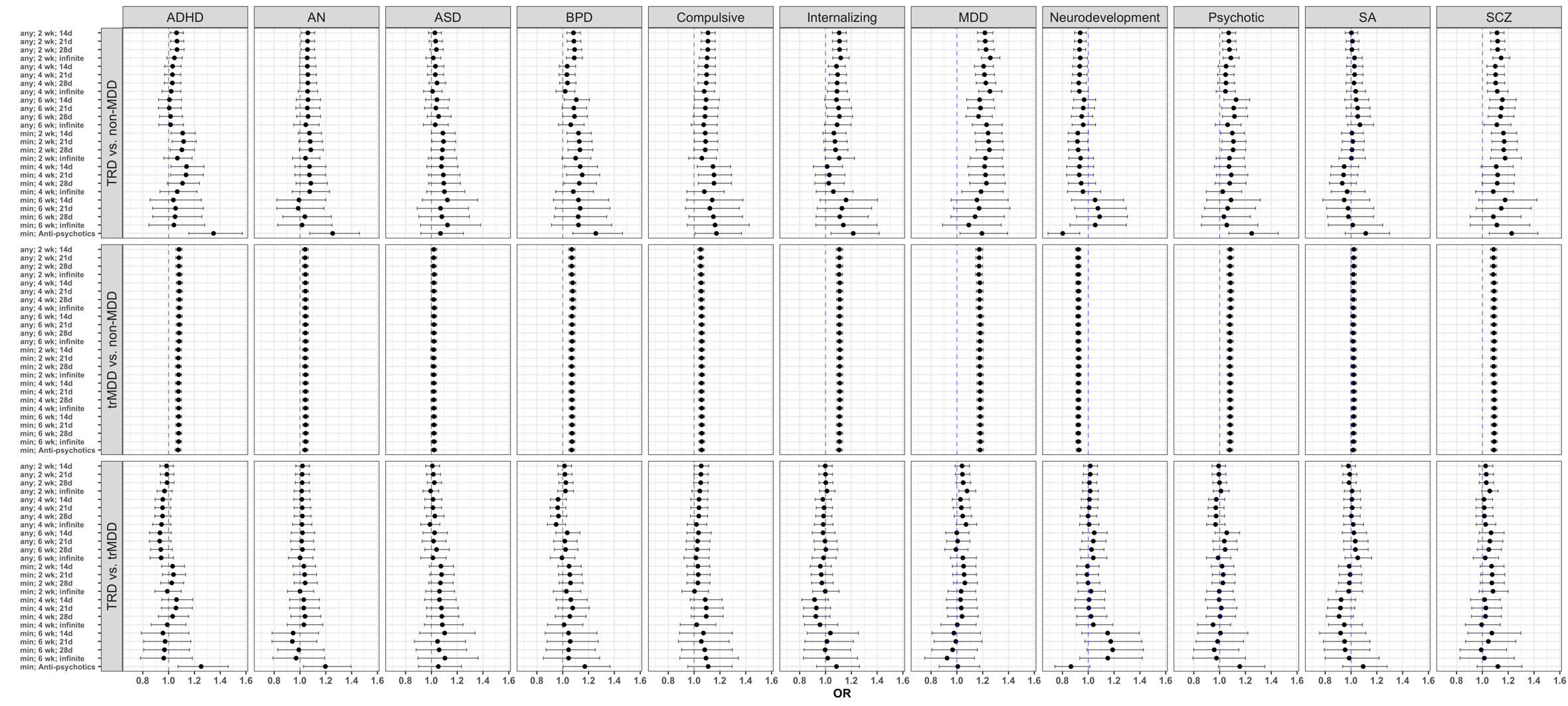


1. **Psychiatric**

**
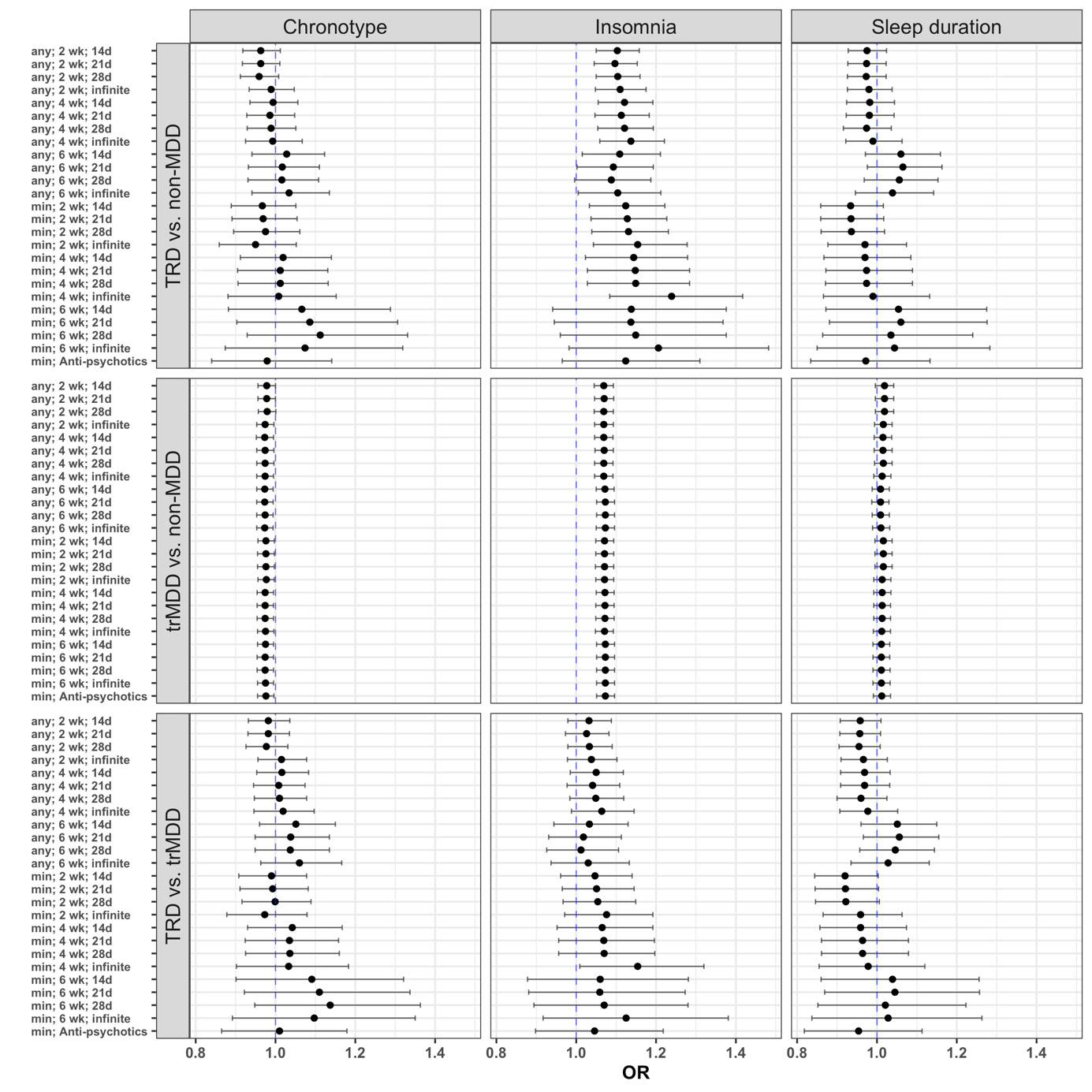

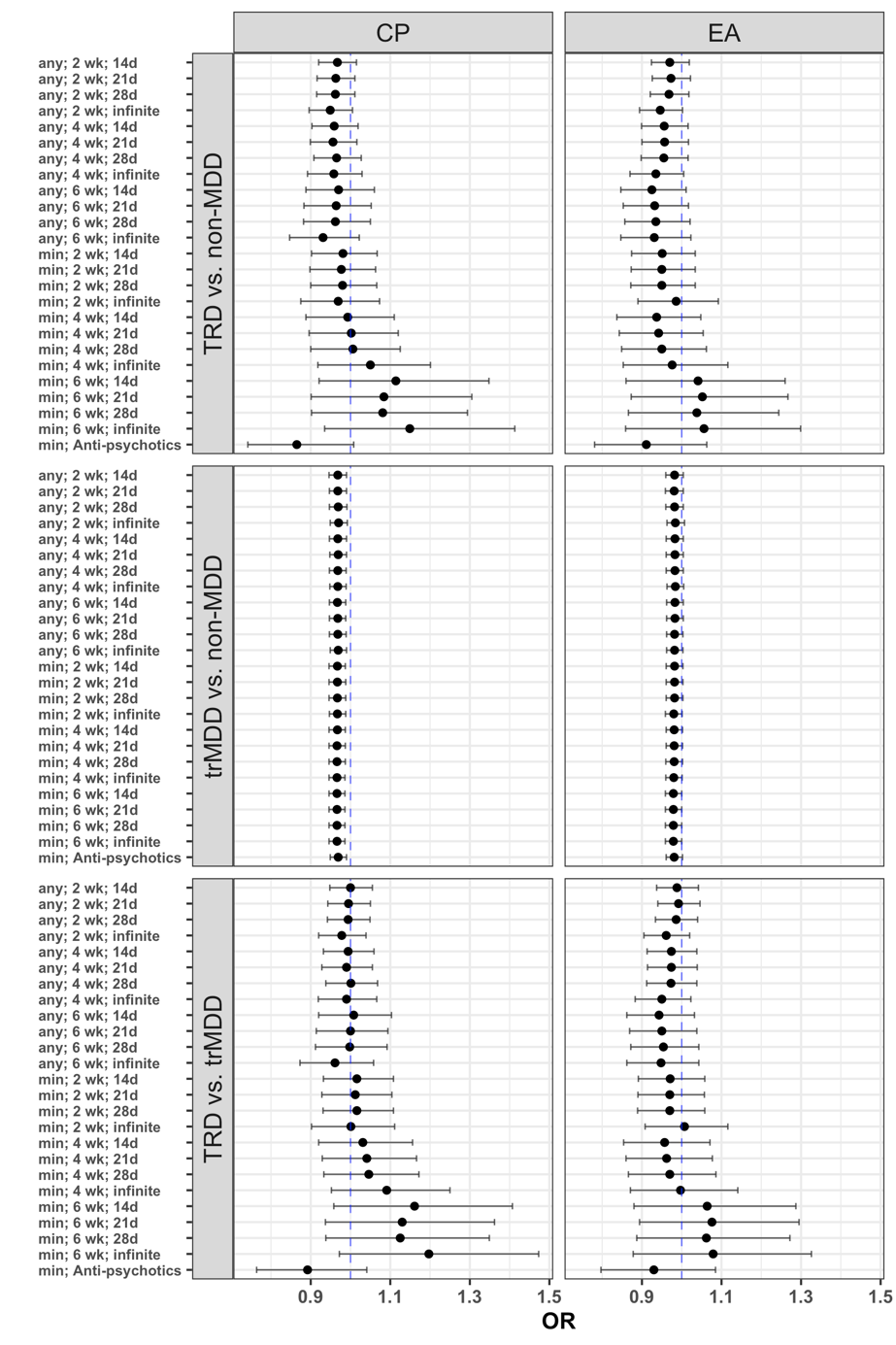
**

**(b) Sleep patterns (c) Education and cognition**

**
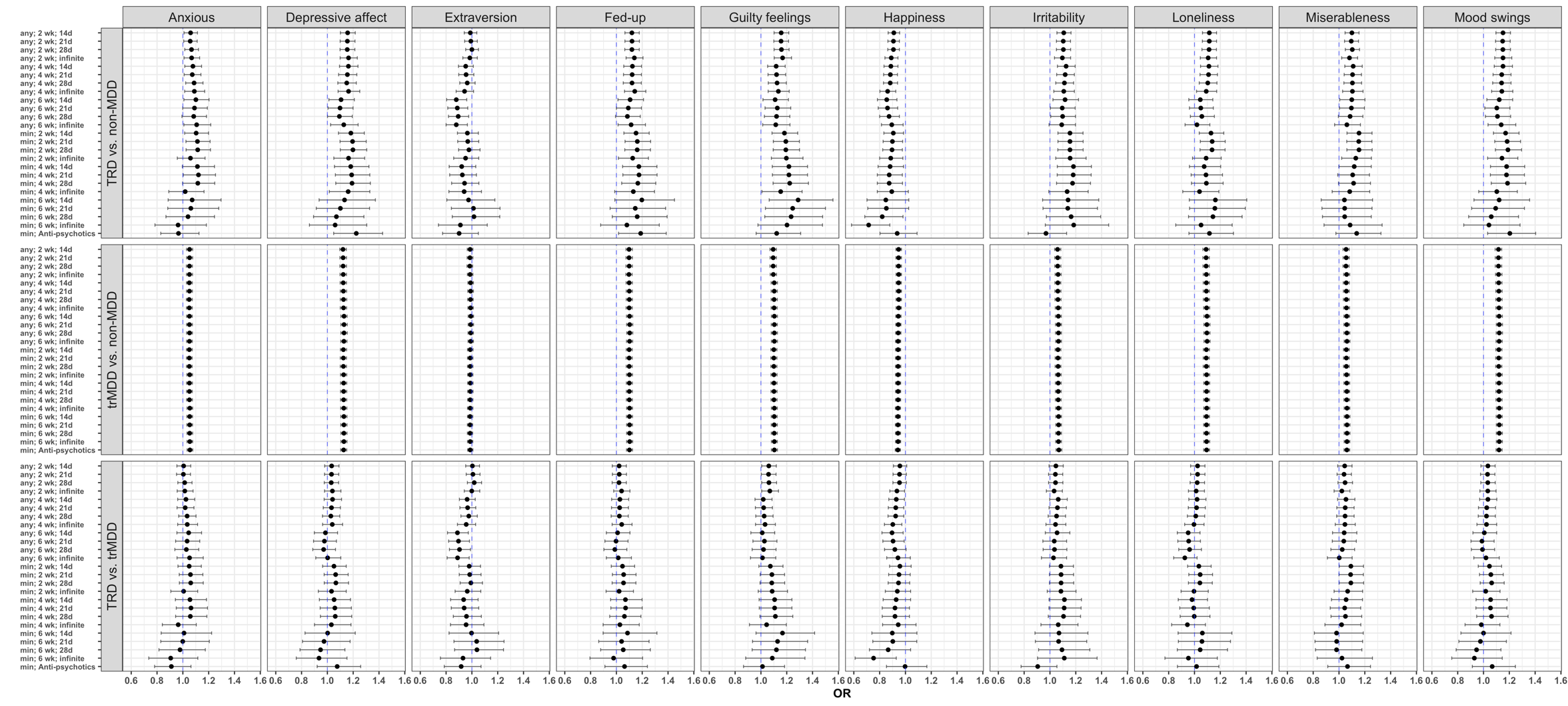
**

**(d) Personality part 1**

**
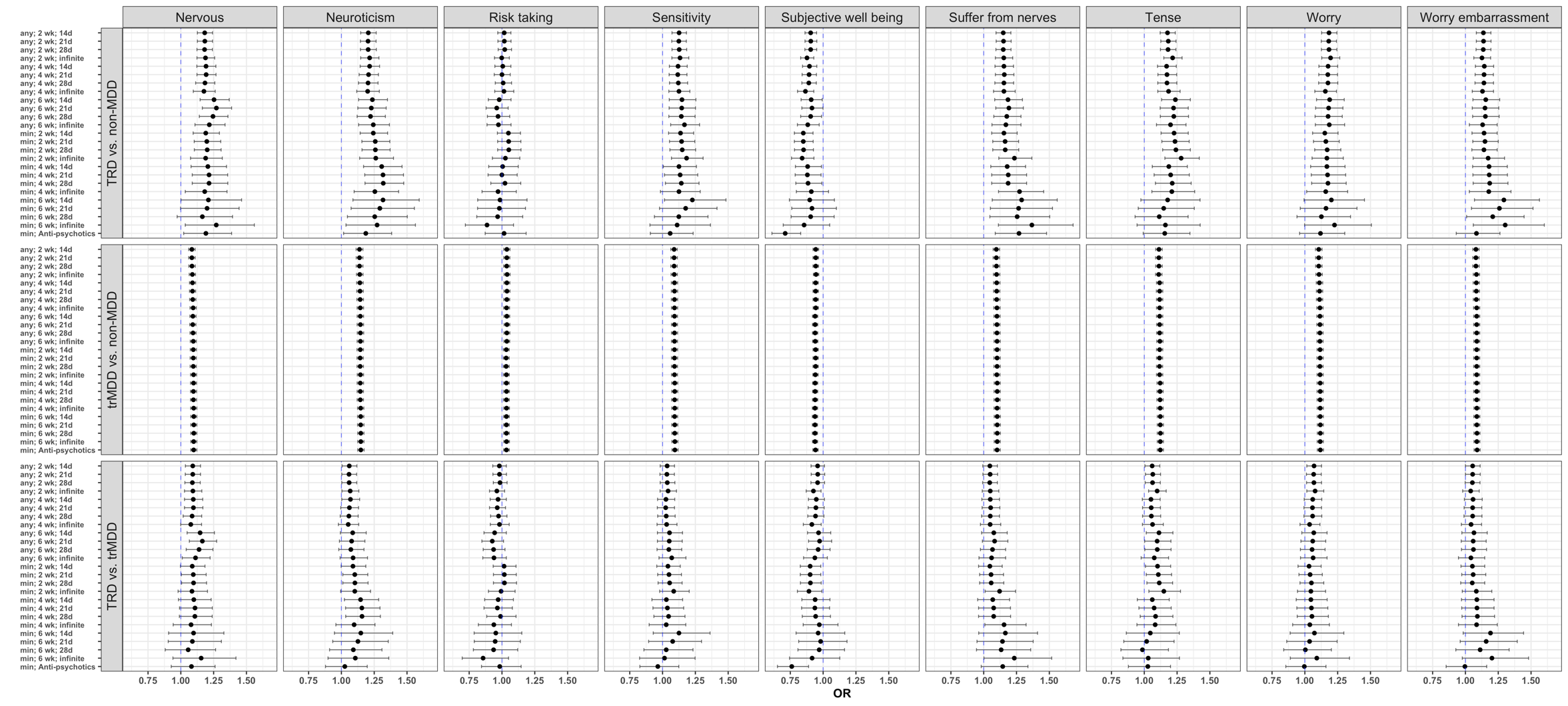
**

**(d) Personality part 2**


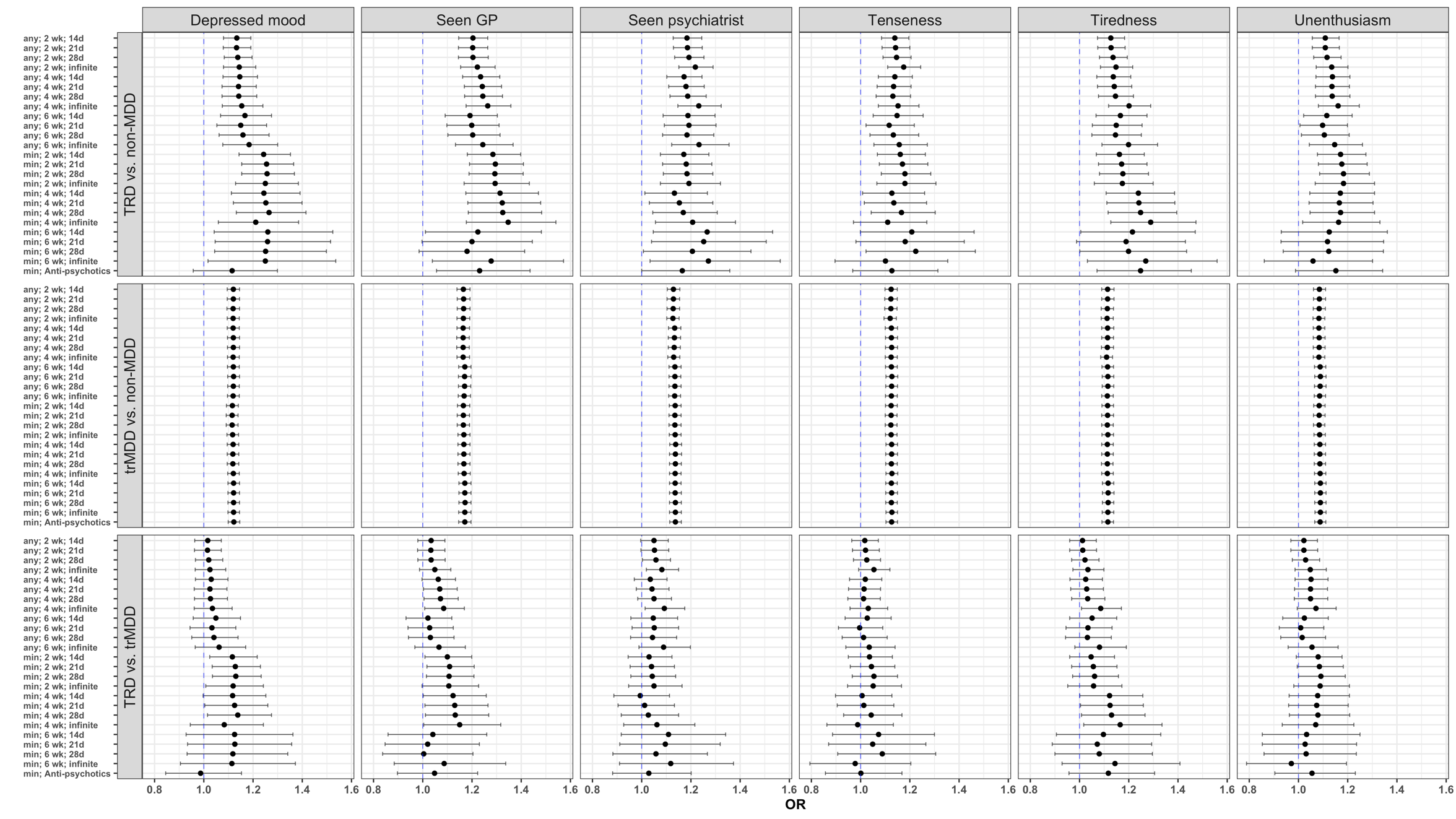


**(e) Temperament**

Supplementary Figure 1. Sensitivity Analyses for different treatment-resistant MDD (TRD) definitions. The forest plots for the association of 41 PGSs of five domains (a)-(e) with TRD vs. non-MDD, trMDD vs. non-MDD, and TRD vs. trMDD among 25 TRD definitions (any or minimal dose; a given duration of ≥ 2, 4, or 6 weeks; interrupted window of < 14, 21, 28, or infinite days; and anti-psychotics add on).


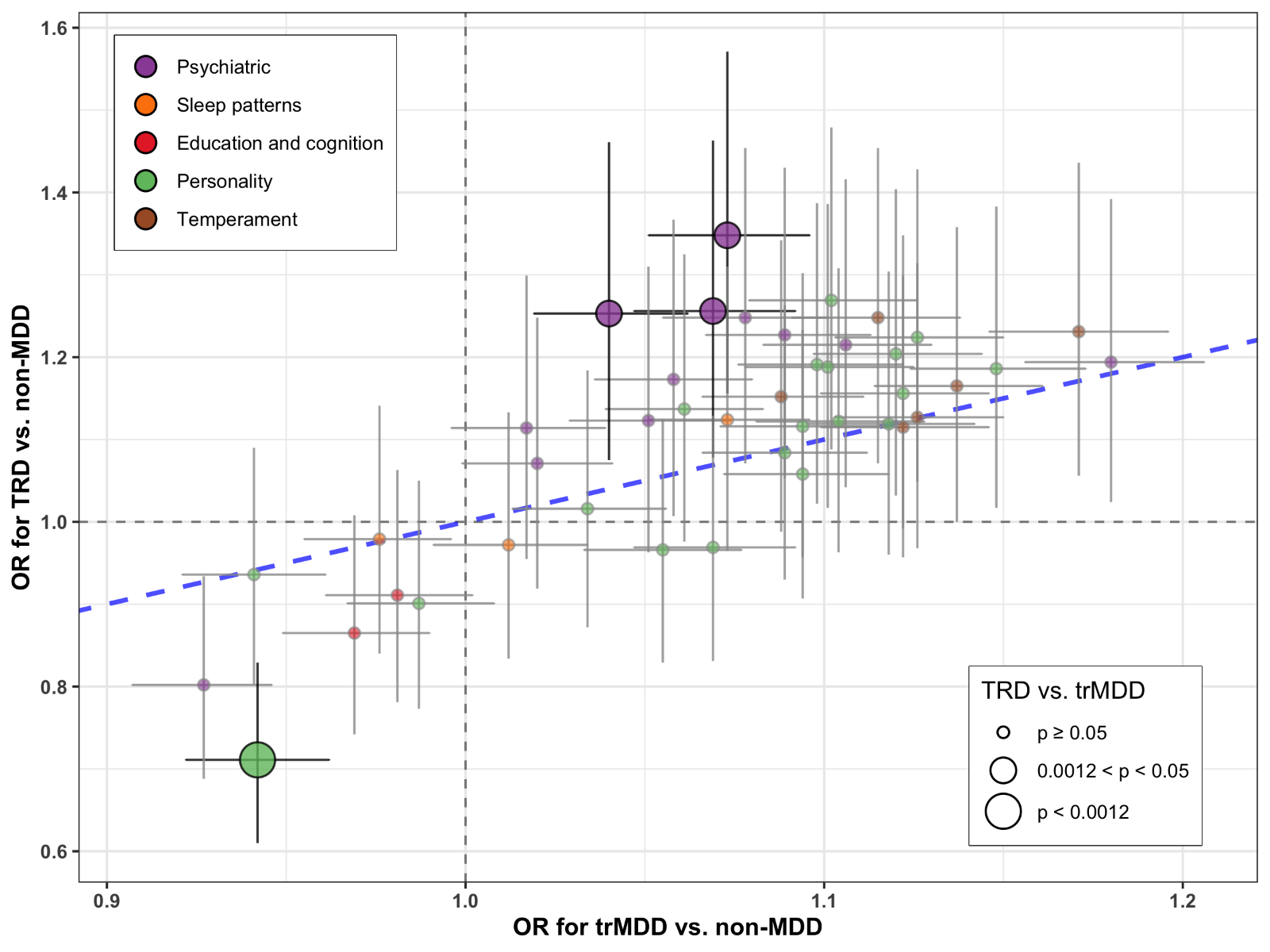


Supplementary Figure 2. Association of PGS with treatment-resistant MDD (TRD) defined by antipsychotics add on within an observation period of 2 years after initiating antidepressant treatment. The scatter plot of the odds ratio (OR), with the corresponding 95% confidence interval, between PGS and TRD vs. non-MDD against the OR between PGS and trMDD vs. non-MDD. PGSs significant associated with TRD vs. trMDD are showed in solid colors.


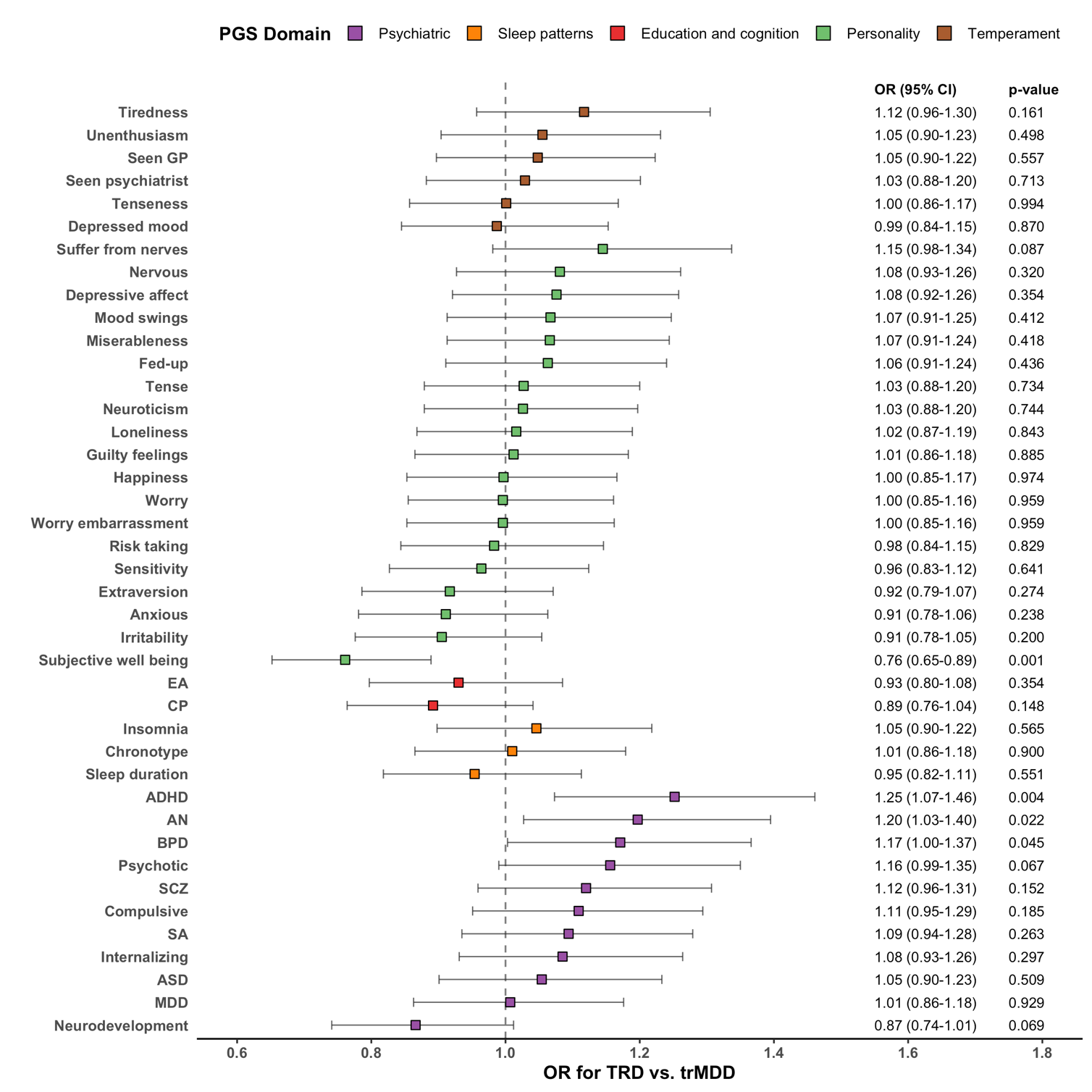


Supplementary Figure 3. Forest plot of the odds ratio (OR) with the corresponding 95% confidence interval between PGS and TRD vs. trMDD. TRD was defined by antipsychotics add on within an observation period of 2 years after initiating antidepressant treatment.


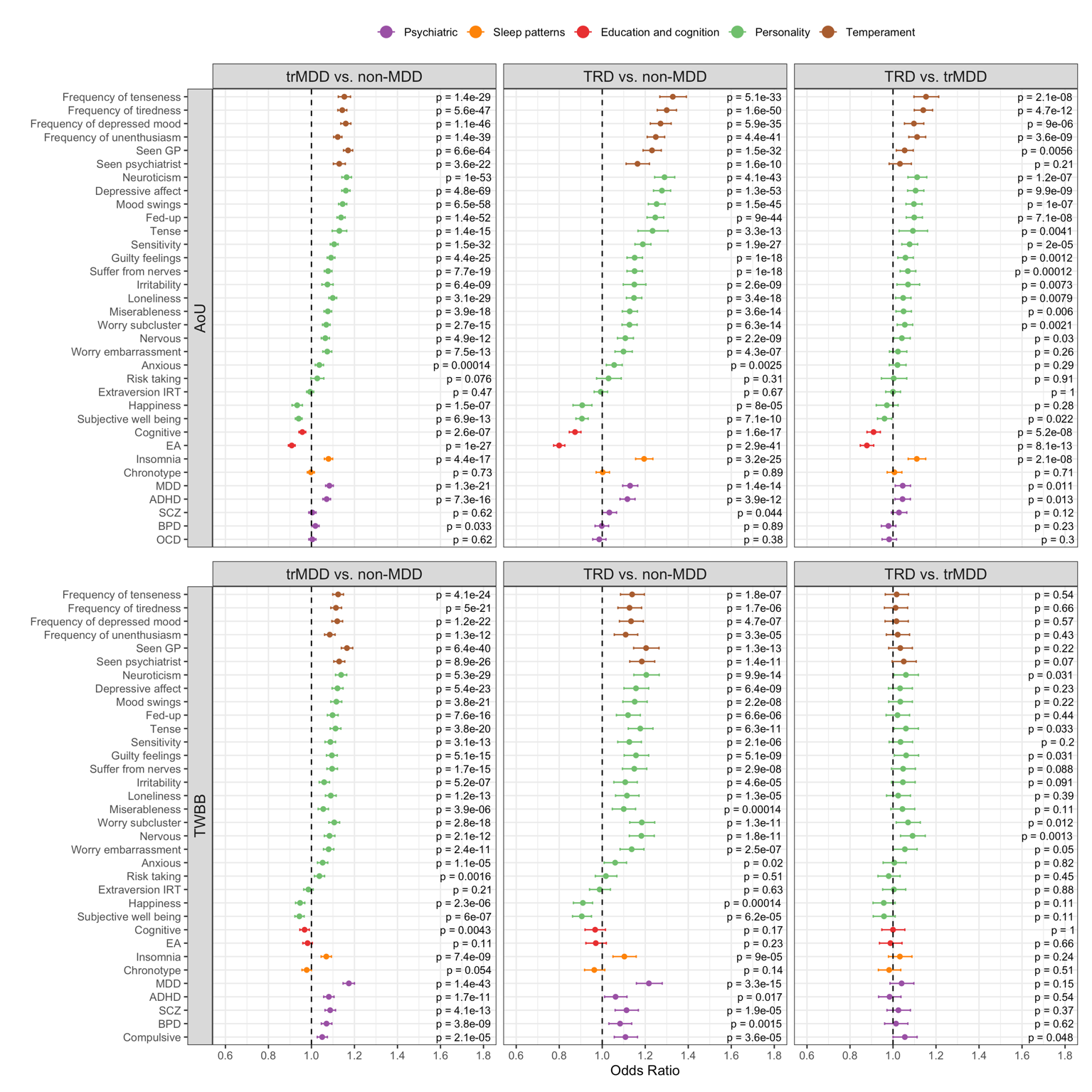


Supplementary Figure 4. Association of PGS with treatment-resistant MDD (TRD) and treatment-responsive MDD (trMDD) in Taiwan Biobank of East Asian populations and in All of US of European populations. Forest plot of the odds ratio with the corresponding 95% confidence interval.
